# Low serum 25-hydroxyvitamin D at critical care initiation is associated with sepsis and morbidity in Dutch critically ill patients

**DOI:** 10.1186/cc14445

**Published:** 2015-03-16

**Authors:** K De Haan

**Affiliations:** 1 Erasmus MC, Rotterdam, the Netherlands

## Introduction

Vitamin D deficiency may frequently occur in critically ill patients and may be associated with sepsis and increased mortality. We therefore evaluated the prevalence of 25-hydroxyvitamin D deficiency in a Dutch ICU, and its relationship with sepsis, morbidity and mortality.

## Methods

We conducted a prospective observational study in a 10-bed mixed ICU. A total of 1,372 patients were admitted between July 2011 and June 2013 including 198 readmissions, of which 940 patients were studied. 25-Hydroxyvitamin D levels were determined within 24 hours after admission. 25-Hydroxyvitamin D levels were judged as sufficiency (>50 nmol/l), insufficiency (30 to 50 nmol/l) and deficiency (<30 nmol/l).

## Results

The prevalence of deficiency and insufficiency was 36% and 38%, respectively. Only 26% of the patients had sufficient vitamin D levels. Vitamin D deficiency is associated with sepsis (*P *< 0.001) at ICU admission. Patients with deficient levels had higher mean APACHE IV scores, 64 versus 52 (*P *< 0.001), and longer length of hospital stay, 12 versus 9 days (*P *< 0.001), respectively, as compared with patients with sufficient levels. Patients with deficient vitamin D levels had an odds ratio for in-hospital mortality of 1.4 (95% confidence interval of 0.84 to 2.29, *P *= 0.2) relative to patients with sufficient vitamin D levels.

## Conclusion

25-Hydroxyvitamin D deficiency frequently occurs in Dutch critically ill patients. Although relating to sepsis, disease severity and morbidity, vitamin D deficiency is not an independent predictor of mortality in these patients, which was otherwise relatively low.

